# “I’m Still on Track”: A Qualitative Exploration of Participant Experiences of a Weight Loss Maintenance Program

**DOI:** 10.3390/healthcare8010021

**Published:** 2020-01-16

**Authors:** Bronwyn McGill, Blythe J. O’Hara, Philayrath Phongsavan, Adrian Bauman, Luke Lawler, Anne C. Grunseit

**Affiliations:** 1 Prevention Research Collaboration, Charles Perkins Centre, Sydney School of Public Health, The University of Sydney, Camperdown, NSW 2006, Australia; 2 The Australian Prevention Partnership Centre, 235 Jones Street, Ultimo, NSW 2007, Australia; 3 Prima Health Solutions, P.O. Box 7468, Warringah Mall, NSW 2100, Australia

**Keywords:** weight loss maintenance, lifestyle program, private health insurance, chronic disease, secondary prevention, thematic analysis

## Abstract

Qualitative evidence of participants’ experiences of real-life weight loss maintenance programs is important for ongoing participant engagement and can inform program improvements. The purpose of this study was to understand how participants account for their engagement with a weight loss maintenance program and the role of the program in their weight management. A qualitative study using semi-structured interviews with 17 participants of a weight loss maintenance program was conducted; common themes were identified using a thematic inductive approach. Many participant narratives incorporated recurrent descriptions of their program experiences as a weight management journey. Our analysis generated four themes: returning to real life as a threat, the personal responsibility imperative, the program supporting agency and the program supporting self-regulation. The program, which provides external support and strategies, overlapped with the context of returning to real life and the personal responsibility imperative. Participant accounts of their journey at this intersection include the program supporting both agency and self-regulation which influences ongoing weight management. The interplay between themes identified and the maintenance program services allows compatibility between participants’ sense of personal responsibility and the program components to help participants to ‘stay on track’ or ‘get back on track’. In providing sufficient structure, opportunities to revisit successful strategies, and accountability, participants are empowered to overcome real-life threats and make positive health choices.

## 1. Introduction

Obesity is a global, chronic and relapsing public health problem in urgent need of prevention and control [1]. As 67% of Australian adults are affected with overweight or obesity [2], secondary prevention measures are important to reduce the individual and community health and economic burden experienced [3]. Intensive behavioural weight loss programs are effective in assisting participants to lose weight [4,5] and are associated with health improvements, including for participants with chronic diseases [6].

For individuals who do lose weight, maintaining weight loss is important for sustaining the resulting short and medium term health and quality of life gains they experience, but is challenging in the long-term [7,8]. Research has shown that lifestyle interventions focusing on dietary and physical activity behaviours [9] and those which extend intervention contact [10] can help participants with weight loss maintenance. One such program is the Long-Term Maintenance Program (LTMP), a low-intensity individualised behavioural support and relapse management program available to individuals who have completed the Healthy Weight for Life (HWFL) program. The HWFL program and the LTMP have been described previously [11,12]. Briefly, the HWFL program is an intensive 18 week weight loss and lifestyle modification program offered to Australian private health insurance members with overweight or obesity and who have osteoarthritis, type 2 diabetes or cardiovascular disease (www.healthyweightforlife.com.au). The program is delivered remotely by a support team of health professionals and focuses on portion-controlled healthy eating (including KicStart^TM^ meal replacements) and recommendations for physical activity. The LTMP extends the contact of the HWFL program to support participants to maintain the weight loss and health benefits attained during the HWFL program with a focus on behavioural support, relapse management and support for weight-related self-monitoring, as well provision of behaviour change resources such as educational materials, recipes and meal replacement products, described in more detail elsewhere [11].

The weight loss maintenance experiences of individuals with overweight and obesity who are part of a lifestyle weight loss maintenance intervention are not extensively reported and participants’ perceptions of the challenges of losing and maintaining weight are not well understood [13]. Qualitative research to date has mostly focused on barriers and facilitators of maintaining weight loss with participants from the general public [13,14,15] or an initial weight loss program [16]. Barriers identified include life transitions, health problems, an absence of accountability and a lack of social support [16]. Key facilitators for weight loss maintenance have been reported to include habit formation, sustained motivation, accountability to others, effective self-regulation, and ongoing professional support, planning ahead, nutrition education and portion control [13,14,15,16]. Systematic review evidence exploring qualitative research on understanding the challenge of weight loss maintenance identified that successful behaviour change for weight loss maintenance required management and resolution of a conflict between overcoming existing habits and meeting personal needs formerly met by obesogenic behaviours [17].

New insights to the experiences of behaviour change for type 2 diabetes participants of a weight management intervention identified how weight loss maintenance behaviour change can manifest over time [18]. This encompassed increasing confidence, changes in attitudes and behaviours needed to adapt from weight loss to weight loss maintenance, an understanding of how new behaviours can affect those of others and a change in participants’ perceptions of themselves. While providing an understanding of the behaviour change experience for type 2 diabetes participants, there is a paucity of qualitative evidence from real-life weight loss maintenance programs including participants with osteoarthritis and cardiovascular disease, and from the Australian private health insurance setting.

An understanding of participants’ experiences of a weight loss maintenance program can provide insights to enhancing their weight loss management. These insights can also inform and facilitate program monitoring, redesign and improvements. As previous qualitative studies have focused on weight loss and weight loss maintenance, the purpose of this study was to explore participant experiences of using the LTMP (as part of an evaluation of the LTMP), in particular:

(a) How do people registering for the LTMP account for their engagement (or lack of engagement) with the different components of the program?
(b) What role does the LTMP play in participants’ HWFL post-program weight management?

## 2. Methods

### 2.1. Researcher Description

All researchers of this exploratory qualitative study were also part of a larger study evaluating the effectiveness of the LTMP. Authors B.M., B.J.O.H., P.P., A.B. and A.C.G. are public health researchers at various professional stages from early to senior career, experienced with evaluating healthy lifestyle interventions in populations with overweight and obesity. The researchers bring to this study expertise in quantitative and qualitative research skills, as well as diverse multi-disciplinary backgrounds in medicine, psychology, occupational therapy and social work. B.M. conducted all telephone interviews and B.M., B.J.O.H. and A.C.G. conducted the analysis. To ensure the research team remained impartial, L.L. as the program service provider was not involved in the data collection or analysis. This study was conducted as part of BM’s doctoral research of weight loss maintenance.

### 2.2. Participants and Recruitment

Recruitment for this study was from participants of the HWFL program who were part of a larger study evaluating the impact of the LTMP involving surveys at the LTMP baseline, at 6 months and at 12 months [11]. Those who completed six months of the LTMP from August to November 2018 were eligible for the current study (*n* = 32). Convenience sampling using rolling recruitment took place from September to December 2018; all eligible participants were invited by mail or email, depending on their preferred method of contact, to take part. The study invitation included study and consent information and written consent was received from 19 participants. Two participants were not able to be contacted and 17 interviews were conducted. Ethics approval was granted by The University of Sydney Human Research Ethics Committee (project number: 2017/760).

### 2.3. Data Collection

Participants of the HWFL program provide their body weight (kg), height (cm) and socio-demographic information (gender, date of birth and residential postcode) to the service provider (Prima Health Solutions) on initial registration for the 18 week program, and body weight on completing the HWFL program. LTMP participants provide their self-reported body weight monthly during the LTMP.

Telephone interviews were conducted by a female researcher (B.M.) with a tertiary allied qualification, trained in qualitative interviewing and thematic analysis techniques, and with previous experience in interviewing weight loss (and other health prevention) program participants. The only previous contact with and knowledge of the interviewer that the participants had was through the survey interviews conducted previously for the evaluation study. A semi-structured discussion guide (see Supplementary Materials) was developed by the research team and reviewed by the HWFL program service provider to explore participants’ experiences of using the LTMP. The discussion guide was based on previous literature of individuals’ experience of weight management programs [19] and drew on knowledge gained from a previous focus group study with HWFL program participants describing their experiences with the program [20]. Participants were asked about their experience with the initial weight loss program as an introduction to the interview, and then about their experiences with the LTMP. The questions explored the support they had received during the maintenance phase, how they had communicated with the support team, the least and most useful components of the maintenance phase and their overall impression of the maintenance phase. Interviews took 25–35 min with an average interview time of 28 min, and were audio recorded and transcribed verbatim.

Participants were allocated a participant identification number, which was used in the coding and analysis to ensure confidentiality. Only the principle researchers had access to the data, and other than the interviewer (B.M). the other researchers did not have access to the key linking the names to the interview data. As data were collected, the interviews and field notes were reviewed informally by B.M. and discussed with co-author B.J.O.H. to inform further data collection; interview questions were iteratively augmented to explore emerging lines of inquiry and confirming data interpretation.

### 2.4. Analysis

Socio-demographic and weight-related variables were analysed descriptively. Age was calculated from date of birth and residential postcodes were used to define Socio-Economic Indexes for Areas (SEIFA) as a measure of social advantage and disadvantage [21] and Accessibility-Remoteness Index of Australia Plus (ARIA) provided a geographical measure of social disadvantage and accessibility to services and opportunities for social interaction [22]. Descriptive data were presented for each participant.

The interviewer checked each transcript for accuracy by listening to the recordings and correcting any errors. Corrected transcriptions were imported to NVivo 11 qualitative analysis software [23]. Two researchers (B.M. and B.J.O.H.) independently coded six interviews to develop an initial coding frame. Common themes, generated from the interview content rather than being predetermined, were identified using a thematic inductive approach [24,25]. Themes were compared for consistency and any disagreements resolved by discussion. The coding frame was subsequently revised and the other 11 interviews coded by B.M. Additional examination by B.M. distinguished recurring themes through an iterative process investigating the reasoning for participant responses to major perceptions discussed. Regular meetings of three researchers (B.M., B.J.O.H. and A.C.G.) were held to ensure transparency of the data analysis. B.M. described themes and discussion with B.J.O.H. and A.C.G. facilitated the identification of dimensions and constructs which were grouped. Resulting themes were reviewed and confirmed against the data, with further refinement ensuring an accurate reflection of participants’ perceptions. Themes were checked across gender and whether participants had maintained weight loss or not. The analysis reached thematic saturation as no new additional information arose despite the different demographic composition of the groups [26].

## 3. Results

### 3.1. Participant Characteristics

Of the 17 participants interviewed, nine were male and eight were female and their average age was 68.1 (±8.1) years. Participants in this study were from across the quintiles of socio-economic disadvantage and 11 participants were from major cities. Ten participants were from the HWFL osteoarthritis program and seven from the HWFL cardiovascular disease program. Characteristics of individual participants are summarised in Table 1.

All participants were affected by either overweight (*n* = 10) or obesity (*n* = 7) at the LTMP baseline. One participant had not lost weight during the HWFL program; 16 participants had lost 5% or more of their starting weight. On average, the participants interviewed lost 9.96 kg (±4.1, range: −19.0 to +1) during the initial HWFL program. At the LTMP baseline, the average weight of participants was 90.6 kg (±18.5, range: 67.7 to 145.0) and their average BMI was 34.5 kg/m^2^ (±6.0, range: 22.5–47.9). On average, participants’ weight change from the LTMP baseline to 6 months (i.e., the time of this study) was +1.2 kg (±3.7, range: −6.5 to +11.3).

### 3.2. Main Findings

Our analysis generated four themes and the results are presented accordingly: Theme (1) returning to real life as a threat, Theme (2) the personal responsibility imperative, Theme (3) the program supporting agency (with four subthemes: experience with weight loss, support and encouragement, realistic expectations and practical resources), and Theme (4) the program supporting self-regulation (with two subthemes: accountability and feedback).

The transition from the initial 18 week HWFL program (or weight loss program) and the subsequent LTMP (or maintenance program) by participants appeared to have been seamless in terms of how they viewed each program. While participants recognised the different aims of each program, they often did not differentiate between these when describing their engagement with the maintenance program. All talked about their experience of the initial weight loss program positively and many described the structure of the program and the support provided by the HWFL team as key elements of their experiences with the maintenance phase.

The narrative of many discussions regarding the maintenance program incorporated recurrent descriptions of ‘staying on track’, going ‘off track’ and getting ‘back on track’. The imagery used by many participants to describe their experiences as a ‘weight management journey’ implied (a) an intended destination and (b) a defined path to reach that destination, albeit with challenges along the way. The ‘destinations’ participants seemed to be trying to reach using the maintenance program were: to maintain the weight loss achieved during the initial weight loss program, or to lose more weight. The ‘track’ travelled to reach their destination was the narrative of their attempts to maintain healthy eating and physical activity habits learned during the HWFL program.

**Figure 1:**
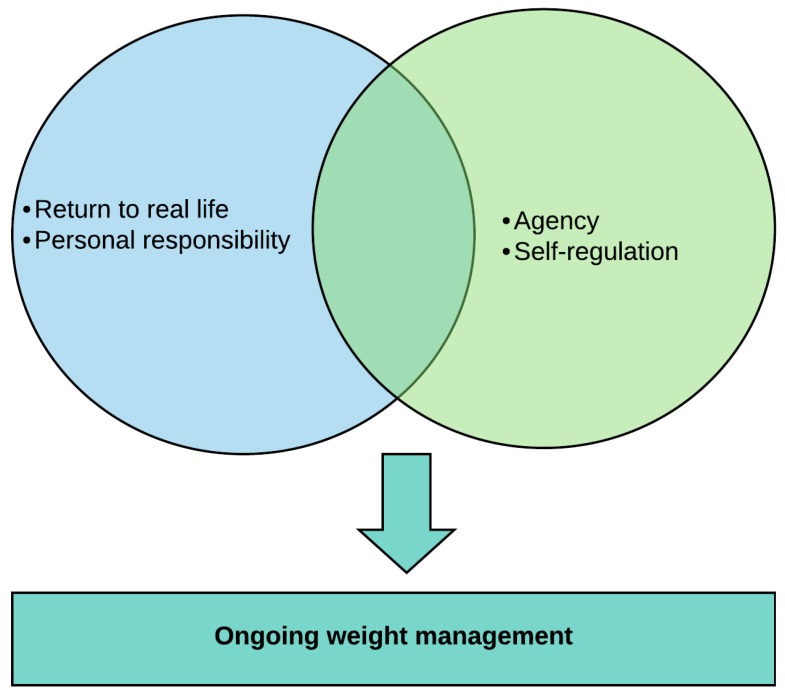
The intersection of themes which shaped ongoing weight management.

Participants’ accounts of the role of the HWFL program and the LTMP in their weight management journey, centred on how the maintenance program kept them on track and helped them back on track should they have deviated from their intended trajectory. The four themes described the means by which participants’ weight management journeys were shaped. The context of returning to real life as a threat (Theme 1) and the personal responsibility imperative (Theme 2, Table 2) intersected with agency (Theme 3) and self-regulation (Theme 4) which are supported by the program, to influence participants’ ongoing weight management (Figure 1, Table 3). As no consistent thematic divergence by gender or weight loss maintenance at six months was noted, themes are presented for the participant group as a whole.

#### 3.2.1. Theme 1: Returning to Real Life as a Threat

Most participants interviewed had successfully lost weight during the intensive HWFL program despite describing difficulties in doing so. All also spoke about challenges and obstacles they had faced during the maintenance program in managing their weight (whether the goal was further weight loss or weight loss maintenance). Threats included reverting to old habits and fading motivation, inferring a sense of a ‘return to reality’ after the intensity of the initial 18 week weight loss program. In some instances, the threat was realised and resulted in them ‘getting off track’ during the maintenance program. Participant quotes illustrate the challenge some participants found in sustaining the focus and effort of the intensive weight loss program when they moved to the maintenance program (Table 2).

As may be seen in Table 2, ‘real-life’ events and situations which threatened to derail participants’ weight management journeys included transitory events such as holidays, celebrations, illness and injury, which were a part of everyday life but endangered participants’ healthy routines and habits needed for their weight loss management. Longer-term threats included family responsibilities and the timing of starting the program in relation to other real-life events. One participant (Participant 11) talked about how the timing of the program worked for them as they felt that the threat of real life ebbed and flowed, and as a result so did their success with their weight management. Hence, the move to weight loss maintenance program signalled a transition from the more intensive and restrictive practices of the initial weight loss program to more sustainable weight loss maintenance practices of the LTMP.

#### 3.2.2. Theme 2: The Personal Responsibility Imperative

Despite the sense that real life threatened their ability to stay on track with their weight management in ways they could not control, many participants felt that the way back was something they themselves were responsible for (Table 2). That is, they felt that they would not succeed passively in progressing along their weight management journey. Ways in which participants felt they could do ‘the right thing’ was by being disciplined and having self-control with sticking to the healthy routines related to portion control and regular physical activity developed during the weight loss program. They also talked about being disciplined with the self-reporting requirements of the maintenance program. Participants believed that they needed to work hard to succeed and that they alone could enact the healthy behaviours necessary to stay on track. Given their investment in a discourse of personal responsibility for their weight management, and the explicit imperative to enact ‘self-control’, it was important to understand how the participants reconciled these with the maintenance program.

#### 3.2.3. Theme 3: The Program Supports Agency

Despite subscription to the personal responsibility imperative, participants still found use for the maintenance program to overcome challenges and threats to their weight management journey (Table 3). According to the interviewees the program was structured such that it fostered a sense of autonomy and control: they knew what they needed to do and felt empowered to do so. The LTMP gave users experiences and a selection of tools which participants could draw on according to their individual preferences. The flexibility of the program to fit into their own life realised a sense of mastery over their weight management journey.

While the initial program is more structured, the maintenance phase offers flexibility and tailoring in relation to the tools participants can draw from as well as frequency and type of contact, with the HWFL team depending circumstances and needs. For example, participants reported that the individualised nature of support offered by the program was key to their weight loss management and as such seemed to facilitate rather than replace personal agency. Based on the participants’ descriptions of how frequently they used the resources and how important they thought they were when describing their weight management, not all program components were of equal value to all participants. However, most related that they felt confident in knowing ‘what’ they needed to do and ‘how’ they needed to do that in order to manage their weight. In our analysis we identified a sense of personal agency in four subthemes. Specifically, participants related their ongoing weight management experience facilitated their feelings of autonomy and control through current and previous weight loss experiences, support and encouragement from the HWFL team, realistic expectations and goals, and practical resources.

##### Experience with Weight Loss

Participants in explaining their success or otherwise made reference to their experiences with weight loss and the lessons they had learned during the weight loss program such as the establishment of new routines and the changing of old habits. These ‘habits’ were primarily healthy eating habits, rather than physical activity despite the latter being mentioned as a contributor to weight management. In establishing new habits and reaping the benefits from them, participants seemed to feel empowered to continue with these behaviour changes during the maintenance phase. As well as demonstrating a sense of agency through developing new habits, there was also a connection between the participant’s experience on the weight loss program with the sense of responsibility to putting his/her knowledge into action.

Regardless of their intended destination, participants expressed a sense of pride, purpose and ownership when discussing their experiences so far with the maintenance phase. The quotes in Table 3 demonstrate how they attributed their weight loss to their own hard work, and as such, inspired them to maintain that loss, or spurred them on to further weight reduction. In this way, although they were being supported by the program, their sense of agency remained intact and combined with the sense of personal responsibility to fuel their self-efficacy to achieve their goals.

##### Support and Encouragement

Although contact with the HWFL team is less intensive for the maintenance phase than the initial program, all participants expressed appreciation of the support available to them during the LTMP. Participants spoke of the importance of not feeling disheartened when the reality of the challenge of long-term weight loss maintenance presented itself and their weight fluctuated over time. Part of managing those threats to their confidence was being able to access support from the HWFL team when they needed it which they found helpful in maintaining the momentum needed for successful long-term weight management.

##### Realistic Expectations

Another way in which the LTMP helped to empower participants to get back on track included setting and reviewing their goals. A component of the initial weight loss program, participants of the maintenance program continued to use goal-setting as part of their ongoing weight management and as a way of keeping their expectations realistic. Some maintenance program participants re-visited their goals with the help of the HWFL team. They reported that the interactive support of the HWFL team helped in having realistic and achievable goals and expectations for weight loss management longer term by having a pragmatic perspective on weight management. In this way, the program supported personal autonomy, providing support to keep their weight management ‘in perspective’.

##### Practical Resources

Some participants discussed how program resources available to them on the initial weight loss program played a central role in their ongoing weight management as part of the maintenance program. For example, some continued to use information provided about portion control, healthy eating, recipes and energy balance in the context of their everyday meal and physical activity routines. The benefit of an information book provided as part of the HWFL program was highlighted by some as an ongoing resource for weight loss maintenance (Table 3).

A key resource offered by both the weight loss and maintenance programs are Kicstart^TM^ very low calorie meal replacement ‘soups and shakes’, which many participants continued to use beyond the initial HWFL program. Although only a few participants continued to use meal replacements on an ongoing and/or everyday basis, many participants reported using them intermittently to self-manage their weight at times when they regained weight, were struggling with maintaining healthy routines or in anticipation of events which threatened their weight management. For example, some participants used meal replacements while travelling away from home, or following special occasions involving a change to their established healthy eating routines.

#### 3.2.4. Theme 4: The Program Supports Self-Regulation

A common thread in our interview narratives was the role of the maintenance program in supporting participants’ self-regulation (Table 3). The program provided surveillance tools which facilitated self- and external-monitoring by the HWFL team, which had the effect of making participants feel accountable to themselves and to the team, as well as eliciting helpful feedback to participants.

##### Accountability

Participants identified feeling accountable to the HWFL team as key in keeping on track or getting back on track with their weight management. It stemmed from the mandatory (i.e., minimum of monthly) self-reporting requirements of the LTMP; knowing that the HWFL team would monitor their weight-related progress as well also seemed to promote a consciousness of where they were with their weight and reflecting on any adjustments which need to be made (Table 3). The feeling of accountability was both pre-emptive, where the thought of the accountability prompted monitoring, and reactive, where the accountability prompted their behaviour.

##### Feedback

Feedback from the maintenance program to participants gave insights into their weight management progress, allowing them to assess whether they were on track in their weight management journeys. Most participants valued personalised responses regarding their self-reported progress measurements for weight and waist circumference from a HWFL team member. They found that as the HWFL team knew what had and had not worked for them during the weight loss program, they were able to provide tailored feedback that reinforced that they were on the right track and if they were not, how best to re-focus their efforts. Another important feedback mechanism mentioned by some participants was that of immediate progress-tracking through online graphs depicting their weight-related trends over time.

The self-monitoring and reporting components of the LTMP set the framework for accountability both to the participants themselves and to the HWFL team which inspired them to attend to their goals and their progress against them and make adjustments where needed. Monitoring also provided a scaffolding for feedback from the service by which they could gauge the impact of their strategies and elicit appropriately targeted reactions from the HWFL team. Thus, self-monitoring yielded both pre-emptive and reactive responses which helped participants feel in control of their weight management journey.

## 4. Discussion

Our study analyses how participants of a weight loss maintenance program account for their experience of the program and the role of the program in their weight management journey. We describe four themes from interview narratives that anchored how participants viewed managing their weight: the threat of returning to real life after the intensive initial weight loss program, a strong sense of personal responsibility for their weight management, the program supporting their personal agency and the program supporting self-regulation to achieve ongoing weight management. The themes, and how they co-exist and overlap, help to build our understanding of the weight management experiences of LTMP participants, and the value it brings. Interestingly, the positive perceptions of the program did not vary by weight loss maintenance status. Therefore, it appears that the value of the program is not wholly contingent upon the outcome of weight loss maintenance, but rather on the service given to the themes described here.

The challenges posed by returning to real life and the discourse of personal responsibility are the context in which LTMP participants experienced their weight management journey. Despite different chronic disease participants, these accounts were similar to qualitative research findings which grouped Finnish type 2 diabetes participants’ lifestyle change experiences into those who battled against threats to their lifestyle change efforts and those who successfully achieved and felt in control of a healthier lifestyle and routine [27]. The third group of experiences in the Finnish study included participants who felt personally responsible for their behaviour but were not able to enact this responsibility, which is only partly consistent with our findings. While participants in our study expressed a sense of responsibility for their weight management as those in the Finnish study, in contrast to some in that study our sample also felt empowered by the program to ‘do the right thing’. The program provided them with the skills to manage their weight in the longer term, in turn supporting their feelings of being in control of their journey.

Participants who expressed responsibility for, and ownership, of their weight management journeys and overcoming any threats to their progress, argued that only they could put in the hard work needed to succeed on the program. This sense of personal responsibility for changing health behaviours and losing weight has been reported elsewhere among Australian individuals with obesity, but for those with severe obesity this encouraged powerlessness as opposed to encouraging empowerment [28]. Participants in our study did not however, describe feeling that they were to blame for, or disempowered by getting off track [28,29]. They claimed to know what they needed to do to get back on track which they attributed to lessons learned during the HWFL program and to the ongoing relapse prevention strategies [30,31] embedded into the LTMP. These findings indicate that for participants in our study, the program empowered them to keep on track with their weight management, despite setbacks.

Although the personal responsibility discourse was ubiquitous, participant narratives also accommodated a need for the program and recognition of the utility of the program; these opposing notions seemed to co-exist with the agency participants felt within the structure of the program [32]. The tension between individual responsibility and the need for external support mechanisms for weight loss maintenance in a similar population has been described previously [20]. This finding also reflects other research findings where reliance on personal responsibility and self-reflection for weight management was found unsuccessful, and participants ‘changed their strategy’ to include more reliance on external surveillance from an intervention/program [27]. Other qualitative research has shown that practitioner help is needed for individuals to feel confident to apply strategies specific to the maintenance of health behaviours [33]. In balancing personal responsibility and agency with external support, the LTMP seems to have met the need for a support mechanism by providing tools to facilitate weight management which are embedded in participants’ experiences. Without absolving participants of personal responsibility, the program allows them to enact it by providing the mechanisms for ongoing weight management.

Rather than locating success or failure with the individual, the maintenance program engendered shared responsibility between the participants and the LTMP. In feeling part of the program, participants felt supported while still maintaining agency with their weight management journey. The maintenance program fostered a collaborative relationship between the participant and the HWFL team, providing conditions that support personal responsibility, thus ‘bridging the divide’ between individualistic and collective responsibility as suggested by Brownell [34]. The development of such a partnership seems key to successful health coaching and facilitating the achievement of health-related goals [35]. According to the health coaching process is the recognition that an individual is the expert in their own situation [36]. While some participants in our study felt that they were ‘doing it themselves’, the reality is that the program is supports agency in line with the purpose of health coaching [37]. Thus, the program seems to become an extension of themselves, with participants not clearly distinguishing between the weight loss and maintenance programs or between their personal responsibility, their personal agency and the program’s responsibility to them.

One of the roles of the maintenance program appears to be that of a perceived safety net, offering participants a set of weight management tools to choose from as and when they see fit. While its ‘fit for purpose’ nature makes it difficult to determine which components contribute to the success (or not) of the maintenance program, the key ingredient according to our data seems to be that it allows flexibility and autonomy. Participants seemed to accept that they would have ‘ups and downs’, but the program had given them agency in managing their weight, including how they manage weight regain or relapses. They draw on program components that worked for them during the HWFL program, using these as relapse prevention or management strategies. For example, the maintenance program promotes self-regulation through self- and external monitoring, feedback and accountability to the HWFL team. In doing so, the program recognises the significance of self-regulation skills as an important predictor of behaviour change maintenance [38] as well as the value of providing multiple strategies for achieving a goal [39].

The intermittent use of meal replacements exemplifies another way that the LTMP supports participants to manage relapses or anticipated relapses in the context of real-life events [40]. The way in which our study participants viewed the role of the LTMP is aligned with research highlighting the importance of (a) recognising that relapses are possible and are in fact, likely, and (b) that self-regulatory approaches suited to an individual’s needs and real-life context should be put in place as a relapse management strategy [33,41]. Other studies have shown that avoiding deprivation and controlled relapses involving planning for and allowing specific situations and special occasions to occur are important for successful weight loss maintenance [14,18,42].

### Strengths and Limitations

Our study considers the perceptions of participants about the role of a weight loss maintenance program in their weight management journey six months after completing a weight loss program. While most qualitative studies related to weight loss maintenance have focussed on a longer follow-up, our study provides insight to participant experiences in the short-mid term. The relatively small sample of participants in our study, although a convenience sample, had a similar proportion of participants who had maintained their weight loss to the cohort from which they were drawn (data not yet published). Participants in our study were older (average age 68 years) than the cohort from which they were drawn (average age 63 years). Findings of this study may not be generalisable to weight loss maintenance in a younger population. A limitation of the study is that it did not include participants who had type 2 diabetes which also limits study generalisability. The reasons for the lack of type 2 diabetes participants is unclear, but the number of LTMP participants who have type 2 diabetes is lower than for osteoarthritis or cardiovascular disease; and the relatively short recruitment timeframe may also be a factor. Our response rate was 53% and therefore, as with all studies relying on voluntary participant, with a higher rate we may have drawn some different opinions [43]. However, the sample did include both men and women and those who did and did not maintain their weight loss. There is a possibility, given the overall positive perceptions of the program that only those who felt positively about their experience consented to participate, and that other themes may be in operation for those who felt less favourably towards the program.

## 5. Conclusions

Our participants’ accounts of managing and maintaining weight loss reflected a complex interplay between the context of real-life threats and a sense of personal responsibility, and the services offered by the LTMP. The program is compatible with participants’ sense of personal responsibility as its components support agency and allow self-regulation in the ongoing weight management progress. In providing sufficient structure, opportunities to revisit successful strategies, and accountability, participants are motivated to overcome real-life threats and make positive health choices. In effect, participants are able enact the personal responsibility for health discourse by picking and choosing from the services available as their weight management circumstances dictate. These findings provide a unique contribution to the weight loss maintenance literature for older program participants. They will help service providers to understand how and why participants value their program. Future research should explore participant experiences with weight loss maintenance programs with younger participants, as well as for participants who withdrew from such programs and who may not have positive perceptions of their program experiences.

## Figures and Tables

**Table 1:** Participant characteristics.

Participant	Gender	Program	Age (Years)	SEIFA ^a^	ARIA ^b^	LTMP Starting BMI ^c^	Initial Weight Loss (%)	6 Months Weight Loss Maintained
1	Female	OA	68	3rd	Inner regional	Obese	−12.2	Yes
2	Male	CVD	72	1st	Major city	Overweight	−10.8	Yes
3	Male	OA	78	4th	Major city	Overweight	−7.8	No
4	Male	CVD	68	5th	Major city	Overweight	−11.0	Yes
5	Female	OA	74	4th	Major city	Overweight	−11.4	Yes
6	Male	CVD	75	2nd	Outer regional	Overweight	−12.3	No
7	Female	OA	65	2nd	Major city	Overweight	−12.4	No
8	Female	OA	79	1st	Major city	Overweight	−16.5	Yes
9	Female	OA	57	2nd	Outer regional	Obese	−6.1	No
10	Male	CVD	66	5th	Major city	Obese	−8.8	Yes
11	Female	OA	50	2nd	Inner regional	Obese	+1.2	N/A
12	Male	OA	68	5th	Major city	Overweight	−10.1	Yes
13	Male	CVD	65	5th	Inner regional	Overweight	−10.5	Yes
14	Male	CVD	63	4th	Major city	Overweight	−11.1	Yes
15	Male	OA	83	5th	Major city	Obese	−16.7	Yes
16	Female	CVD	67	2nd	Inner regional	Obese	−5.4	Yes
17	Female	OA	76	4th	Major city	Obese	−7.7	Yes

^a^ SEIFA (Socio-Economic Indexes for Areas) provides the general level of socio-economic disadvantage of all people in that area; 1st quintile (most disadvantaged) and 5th quintile (least disadvantaged). ^b^ ARIA (Accessibility-Remoteness Index of Australia Plus) is calculated and based on the road distance from a locality to the closest service centre. ^c^ Overweight (25–29.99 kg/m^2^) and obese (≥30 kg/m^2^). LTMP, Long Term Maintenance Program; BMI, body mass index.

**Table 2:** Quotes supporting the context of weight loss maintenance for the participant.

**Returning to Real Life as a Threat**
*It* [the maintenance program] *hasn’t been quite as intense or as often as the original 18 weeks. It’s sort of slackened off a little bit, and I’ve sort of slackened off with the reports too. But I’ve got to get back into doing it weekly and make sure everybody knows how I’m going.* (Participant 2)
*I’ve found the maintenance* [program] *a little bit harder, because—it’s just a bit of a change to the consistency from that first stage* [weight loss program]. *But when I had to maintain it, it gets a little bit more difficult and I found that my weight would creep up a little bit and go down and creep up … I certainly do [think that the maintenance phase is helpful], because it isn’t just stopped at the end of that initial phase. I don’t think I could have kept as strict as I have been, I honestly don’t … I think the thought that that maintenance phase is there and that I can continue, I think that’s very, very helpful.* (Participant 5)
*… these distractions are going to happen no matter what I want to do or what you want me to do … I just knew it was going to happen. It always does on the trips we go away on. And they [HWFL team] advised me to try to do the right things and all that sort of stuff, but I just knew it wasn’t going to occur when I was with so many people and eating so much good food in France and cheeses and wines.* (Participant 12)
*… sooner or later, your brain goes, “Hang on. Life is for living. You’re not in prison, where you keep denying yourself.” Admittedly, I don’t have as many sweets as I had in the past. I’m very particular with what I have now. I just wish my mum would understand. Being Italian, stuffing food in your kid’s face … I keep telling her that, “Look, I can’t eat as much as I used to, all right?”.* (Participant 14)
**The Personal Responsibility Imperative**
*But then I just, I said to my husband, ‘well there is no point in getting upset over it’, I said ‘I just have to work at getting back on track again and aim to get a few more kilos off again’ … it’s the discipline thing … nobody can do it for you, that’s the thing, if you want to lose weight yourself, you’ve got to do it yourself, you’ve got to do the hard yards.* (Participant 7)
*Well, like I said, my understanding of this program is to retrain your brain to re-surface information that you’ve got that’s at the back of the hard drive, if you like, that hasn’t been used for ages, and its self-control. As I’ve said to many people, “The only person who can change things is yourself. You can’t abdicate that responsibility to someone else and expect Healthy Weight for Life or anybody else to do it for you.” I’m the only person who can do it.* (Participant 14)
*I found it challenging. Not anything really to do with the program, I guess it was more in my life, which was my biggest hurdle. And when I mean my life, just my responsibilities. I went great for the first part of the program and you do need to make lifestyle changes and those changes after the first phase really just weren’t fitting into my life and my responsibilities.* (Participant 11)

**Table 3:** Quotes underpinning the themes of agency and self-regulation.

**The Program Supports Agency**	
*I just think that it’s a wonderful program for people that either are working or can’t get to the gym and do those normal things, it gives you the tools and knowledge, probably to self-motivate yourself and to show you that there are nice foods out there … don’t have to live on carrot sticks and yoghurt.* (Participant 11)	
Experience with weight loss	*Well, it [the HWFL program] sort of gives you a routine to follow. And we’re creatures of habit. If we can develop a routine that makes everything flow well and you get the benefit of weight loss and your blood pressure coming down … it’s well worth the effort … I know I can do it. I just have to do it. And I’m confident if I keep talking to the team that they will keep encouraging me and they’ll keep me on the track too.* (Participant 2)
	*I have to remind myself that, hang on, you’ve put in all these hard yards … a lot of sacrifice … you’re not going to fall off the wagon now.* (Participant 14)
Support and encouragement	*But I told myself that I got to the weight that I wanted to be, and then I actually do go up and down on that, but I … it was only about a month ago, I actually rang … and had a motivational conversation with them … that helped me get back on track.* (Participant 1)
	*I do think the maintenance phase is worth it, and I think the support that you get from them, and if it’s only a text every few weeks, at least you know they are still keeping track of you, and you can talk to them if you need to. They are actually really good when you talk to them, not at all judgemental … they’re very encouraging.* (Participant 17)
Realistic expectations	*Basically in my mind … I had my weight goal, and … I can still do better than that and that was, that was a healthy goal. So they [HWFL team] basically ran through “what am I doing?” by way of menus and exercise … by astute questioning they whittled it down to one or two areas and—drew me out on what I should do next. There was an email follow up which went to the re-scheming of goals … you knew where you stood.* (Participant 4)
Practical resources	*I’ve got this manual which is the absolute heart and soul of the program … I know a lot about what I’m doing … understand what I’m doing and what I need to do.* (Participant 6)
	*It covers … the whole spectrum of things, such as the types of foods that you eat, the portion sizes that you eat and the importance of combining it with exercise … it is a very complete book of information to achieve your goal.* (Participant 12)
	*… we went out to a birthday party the other weekend … I enjoyed myself … okay, today I’m going to start the day with a shake and just get back to a nice easy lunch and things like that … and just get back to the program. Refer to the book, look for various food ideas that we can use … that’s been great.* (Participant 15)
**The Program Supports Self-Regulation**	
Accountability	*I’ve tried to lose weight before and you just lose momentum and think, “Gosh, I can’t do this all the time.” By the fact that they’re* [HWFL team] *there, and that I’m kept to a schedule, even if it’s monthly, already I’m thinking I’ll need to report soon.* (Participant 5)
	*It’s one of those things that it’s very easy to get out of control if you don’t watch these things. So by monitoring your weight and waist measurements and a few things like that, it’s a constant reminder, am I doing the right thing? Am I achieving my goals?* (Participant 15)
Feedback	*And my commitment to it is to do that every single week to make sure that I’m reminded of what I’m doing … and that I get a bit of feedback through the program … But mainly see my weight on the graph that they produce every week … to make sure I’m still on track … if those graphs disappeared, and the commitment to [reporting routinely] … if that disappeared, I’m probably more likely to stray.* (Participant 12)

## Supplementary Materials

The following are available online at https://www.mdpi.com/2227-9032/8/1/21/s1, File S1: Discussion guide.
