# Supplementary material for: “I’m Still on Track”: A Qualitative Exploration of Participant Experiences of a Weight Loss Maintenance Program
Source: Healthcare (Basel). 2020 Jan 16;8(1):21. doi: 10.3390/healthcare8010021 (PMC7151193; doi:10.3390/healthcare8010021)
Supplement: Supplementary file 1 [file healthcare-08-00021-s001.pdf]

healthcare

Article

Supplementary Materials: "I'm still on Track": A Qualitative Exploration of Participant Experiences of a Weight Loss Maintenance Program

Bronwyn McGill [1,2,*], Blythe J O'Hara [1], Philayrath Phongsavan [1], Adrian Bauman [1,2], Luke Lawler [3] and Anne C Grunseit [1,2]

[1] The University of Sydney, Sydney School of Public Health, Prevention Research Collaboration, Charles Perkins Centre, Camperdown, NSW 2006, Australia;

[2] The Australian Prevention Partnership Centre, 235 Jones Street, Ultimo, NSW 2007, Australia

[3] Prima Health Solutions, PO Box 7468, Warringah Mall, NSW 2100, Australia;

* Correspondence: bronwyn.mcgill@sydney.edu.au

Received: 17 December 2019; Accepted: 14 January 2020; Published: date

S1: Discussion Guide for HWFL Maintenance Phase Participant Experiences Interviews

Introduction

Thank you for talking to me today. The purpose of our interview is to gather your thoughts and opinions on your participation in the Healthy Weight for Life Maintenance Phase so far. Before we start, I would like to let you know that:

- The interview will last for about 30-45 minutes.
- Whatever you discuss during this interview will be kept confidential and your real name and details will not appear in any of the results. So please be as open and honest as you can be when responding to the questions.
- I will record the interview and the recording will be transcribed. This helps to ensure that what you say is accurately documented.

[*Note to interviewer: Check that consent is completed.*]

Experiences of the Healthy Weight for Life Program

We will start by getting your comments on the Healthy Weight for Life Program that you completed before starting on the Maintenance Phase.

1. How did you find the program overall? Was it worth doing?

2. What did you like most about the program?

3. Were there parts of the program that you didn't like?

Experiences of the Maintenance Phase of the Healthy Weight for Life Program

Now, we'll move on to talking about the Maintenance Phase of Healthy Weight for Life.

4. How have you found **the support** you have received so far during the Maintenance Phase?

Prompt for information regarding:

- *Type of support received*
- *Frequency of contact with HWFL support team*
- *Usefulness of support received*

5. Were there **any resources** provided to you during the Maintenance Phase?

Prompt for information regarding:

- *Type of resources*
- *Usefulness of resources*

6. Have you continued to use **the soups and shakes** during the Maintenance Phase?

Prompt for information regarding:

- *Why have they used or not used soups and shakes*
- *Have they bought them outside of the program or only through Prima Health?*
- *Have they found the discount useful?*

7. How have you used **the HWFL portal** during the Maintenance Phase?

Prompt for information regarding:

- *Whether they access resources through the portal*
- *How often they have provided self-reported measurements*
- *Other uses of the portal*

8. What have you found **the least useful component** of the Maintenance Phase?

Prompt for information regarding:

- *What has not worked well for their weight loss maintenance*
- *What they have found difficult with the delivery of the Maintenance Phase*
- *What they have found difficult with adhering to the Maintenance Phase*

9. What you found **the most useful component** of the Maintenance Phase?

Prompt for information regarding:

- *What has worked well for their weight loss maintenance*
- *What they found worked well with the delivery of the Maintenance Phase*
- *What they found worked well with adhering to the Maintenance Phase*

10. What is your **overall impression** of the Maintenance Phase?

Prompt for information regarding:

- *How useful has the program been in maintaining weight loss*
- *Would they recommend the Maintenance Phase to others wanting to maintain weight loss*
- *Any other comments about the Maintenance Phase*

Conclusion of Interview

Thank you again for talking to me. We will combine your comments and ideas with those we gather from other participants and that should give us a good picture of what participants think of the Maintenance Phase.
